# Evaluation of Postural Sway in Post-stroke Patients by Dynamic Time Warping Clustering

**DOI:** 10.3389/fnhum.2021.731677

**Published:** 2021-12-03

**Authors:** Dongdong Li, Kohei Kaminishi, Ryosuke Chiba, Kaoru Takakusaki, Masahiko Mukaino, Jun Ota

**Affiliations:** ^1^Department of Precision Engineering, School of Engineering, The University of Tokyo, Tokyo, Japan; ^2^Research Into Artifacts, Center for Engineering (RACE), School of Engineering, The University of Tokyo, Tokyo, Japan; ^3^Division of Neuroscience, Department of Physiology, Asahikawa Medical University, Asahikawa, Japan; ^4^Department of Rehabilitation Medicine I, School of Medicine, Fujita Health University, Toyoake, Japan

**Keywords:** clustering, dynamic time-warping, post-stroke, postural sway, standing posture

## Abstract

Post-stroke complications are the second most frequent cause of death and the third leading cause of disability worldwide. The motor function of post-stroke patients is often assessed by measuring the postural sway in the patients during quiet standing, based on sway measures, such as sway area and velocity, which are obtained from temporal variations of the center of pressure. However, such approaches to establish a relationship between the sway measures and patients' demographic factors have hardly been successful (e.g., days after onset). This study instead evaluates the postural sway features of post-stroke patients using the clustering method of machine learning. First, we collected the stroke patients' multi-variable motion-capture standing-posture data and processed them into *t* s long data slots. Then, we clustered the *t*-s data slots into *K* cluster groups using the dynamic-time-warping partition-around-medoid (DTW-PAM) method. The DTW measures the similarity between two temporal sequences that may vary in speed, whereas PAM identifies the centroids for the DTW clustering method. Finally, we used a *post-hoc* test and found that the sway amplitudes of markers in the shoulder, hip, knee, and center-of-mass are more important than their sway frequencies. We separately plotted the marker amplitudes and frequencies in the medial-lateral direction during a 5-s data slot and found that the post-stroke patients' postural sway frequency lay within the bandwidth of 0.5–1.5 Hz. Additionally, with an increase in the onset days, the cluster index of cerebral hemorrhage patients gradually transits in a four-cluster solution. However, the cerebral infarction patients did not exhibit such pronounced transitions over time. Moreover, we found that the postural-sway amplitude increased in clusters 1, 3, and 4. However, the amplitude of cluster 2 did not follow this pattern, owing to age effects related to the postural sway changes with age. A rehabilitation doctor can utilize these findings as guidelines to direct the post-stroke patient training.

## 1. Introduction

A stroke is mainly caused by a lack of oxygen when the brain's blood flow is interrupted by a blockage (i.e., cerebral infarction, CI) or an artery rupture (i.e., cerebral hemorrhage, CH). Stroke patients tend to inherit an irregular postural sway during quiet standing (Chern et al., 2010), which increases the risk of falling. In this regard, evaluation of their quiet standing postural sway is essential.

Researchers have evaluated the quiet standing postural sway of post-stroke patients for many years. However, the postural sway features (e.g., sway amplitude) of the patients can be different because of different patient demographic factors (e.g., days after onset, age, and the influence of the level of damage in lesion regions) (Bansil et al., 2012; Cho et al., 2014; Halmi et al., 2020). Unfortunately, the relationship between postural sway features and patient demographic factors has not been well-studied. Some researchers focused only on sway amplitude. For example, Mizrahi et al. (1989) measured and analyzed the bilateral forces of the supporting limbs of stroke patients and found that they had significantly higher sway activity compared with normal controls. In the anterior-posterior and medial-lateral (ML) directions, (Wang et al., 2017) found that stroke patients had a more pronounced center-of-pressure (COP) sway than healthy people. Paillex and So (2005) demonstrated that temporal patterns of the difference between the COP and center-of-gravity could be characterized differently for healthy subjects and patients. Some researchers revealed greater sway activity in hemiplegic subjects compared with normal controls (Mizrahi et al., 1989).

Furthermore, machine-learning methods (e.g., multivariate time-series clustering) can find postural sway features using complete time-series data. For Parkinson's disease, Das et al. (2011) explored the motor symptoms of patients using a motion-capture system and a support vector machine. However, only a few studies have thus far analyzed the differences in post-stroke patients' quiet standing postural sway. Furthermore, there is no consensus with regards to the best method or feature set for analyzing motion-capture data to understand and assess post-stroke postural sway.

Hence, this study evaluates postural-sway features of post-stroke patients using a motion-capture system with a multivariate time-series machine-learning clustering technique.

The remainder of this paper is organized as follows. Section 2 reports the method of clustering and parameter distribution calculation. Section 3 presents the results. Section 4 discusses the implications of the results. Finally, we present the conclusions in section 5.

## 2. Methods

**Figure 1:**
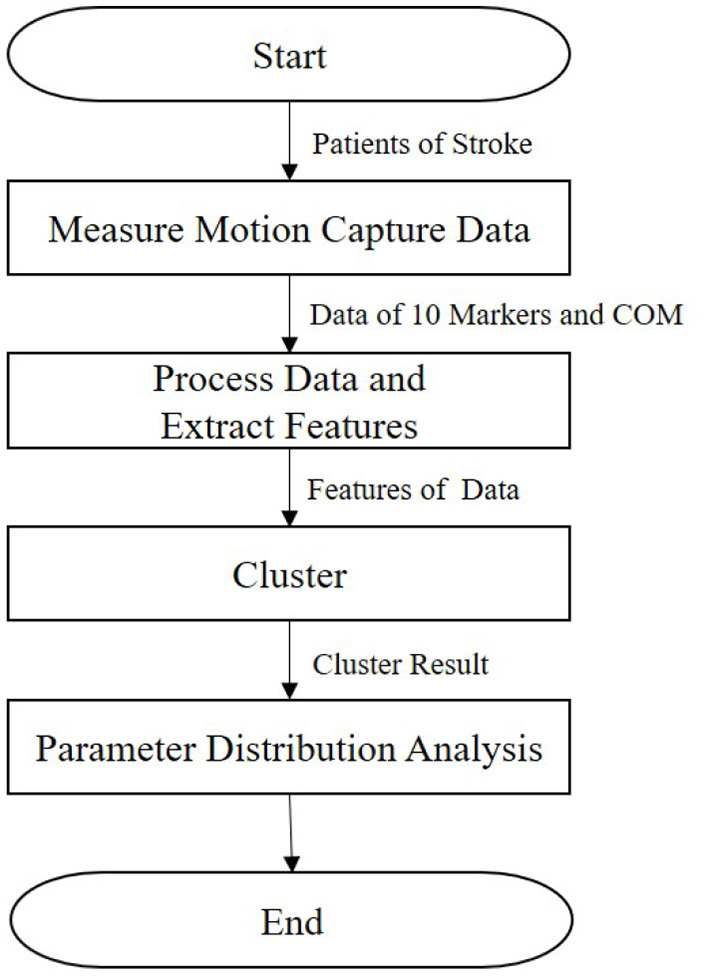
Workflow of the methods used in this study.

In this section, we describe our research method (Figure 1). First, the kinematic posture data of patients are measured, as detailed in section 2.1. Then, we extract features, as presented in section 2.2. Next, the clustering method is described in section 2.3. Finally, we calculate the parameter distribution, as detailed in section 2.4.

### 2.1. Motion-Capture Measurement

#### 2.1.1. Study Population

Fujita Health University recruited the study subjects for our research. According to the agreement approved by the University Ethics Committee, all subjects provided informed written consent (HM18-467). We hired 10 male subjects. Five were CH, and five were CI; their statistical information is shown in Table 1. We added new data to previous research results (Li et al., 2021), including ages, days after onset, and hemiplegia. In our previous work (Li et al., 2021), we assumed that CH and CI presented differences in standing posture and leveraged a support vector machine for classification. However, in this paper, we evaluate the postural sway of post-stroke patients in a quiet standing position via clustering.

**Table 1:** Subject information.

**Name index**	**Disease**	**Age**	**Days after onset**	**Hemiplegia side**
CH1	Cerebral hemorrhage	54	1108/1150	Right
CH2	Cerebral hemorrhage	48	122/129/136/143	Left
CH3	Cerebral hemorrhage	59	51/58/65/79/93	Left
CH4	Cerebral hemorrhage	63	520/527/534	Either
CH5	Cerebral hemorrhage	54	315/340/358	Right
CI1	Cerebral infarction	52	74/88/109/176	Right
CI2	Cerebral infarction	75	132/147	Left
CI3	Cerebral infarction	80	69/80	Right
CI4	Cerebral infarction	63	91/108	Left
CI5	Cerebral infarction	69	992/1007/1020/1051	Right

#### 2.1.2. Patient Data Collection

**Figure 2:**
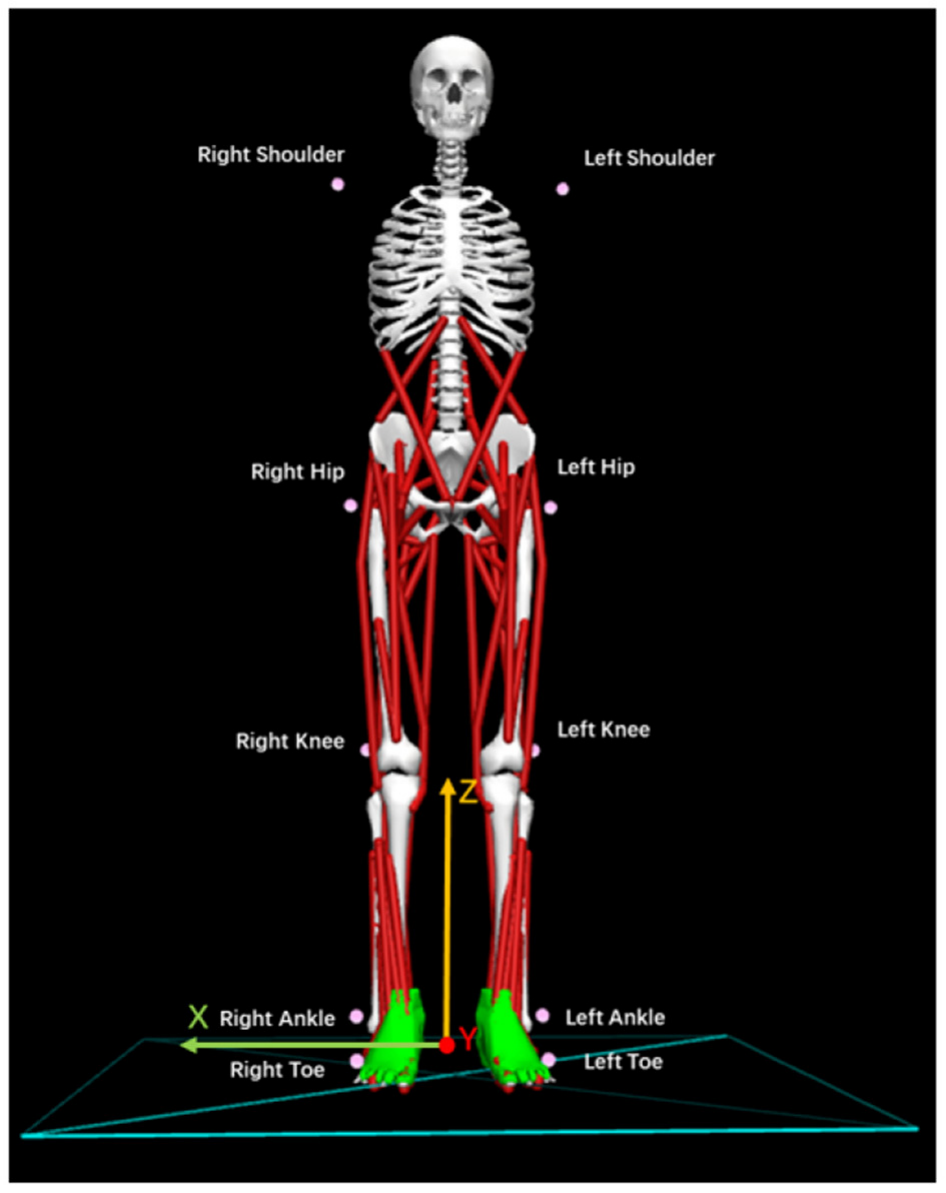
Location and names of markers attached to the patients. Ten pink markers were attached to the body symmetrically.

A 3-dimensional (3D) motion analysis system, KinemaTracer© (KISSEI COMTEC, Matsumoto, Japan), was used to precisely measure the quiet standing posture of post-stroke patients. The system hardware leveraged one recording/analyzing laptop and four charge-coupled-device cameras arranged around a standing platform on a level floor without a handrail. As shown in Figure 2, we attached the markers (30-mm diameter) to the acromion on both sides of the subject: the hip joint (positioned on the line connecting the superior anterior iliac spine and greater trochanter at 1/3 of the distance from greater trochanter), the knee joint (the AP midpoint of the lateral epicondyle of the femur), the ankle joint (exterior), and the toes (fifth metatarsal head). The measurement sampling frequency was 60 *Hz* for 30 *s*. A more detailed description of our methodology and mathematical formulas for data collection can be found in Matsuda et al. (2016) and Li et al. (2021).

### 2.2. Data Processing and Feature Extraction

#### 2.2.1. Kinematic Collected Variables

In this study, we utilized 33 kinematic variables extracted from the 3D motion analysis system for post-stroke patients' postural-sway evaluation. The kinematic variables contain 3D displacements (X-axis, Y-axis, Z-axis) of the center of mass (COM) and 3D displacements of 10 markers during the 30-s period. Hence, we collected 33 kinematic variables.

#### 2.2.2. Feature Extraction

**Figure 3:**
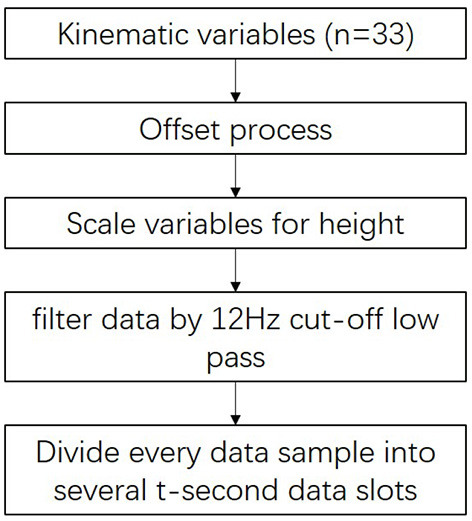
Methodology framework of the feature extraction described in section 2.2.

We extracted features for use when clustering post-stroke patient data with high performance. The methodology of this process is described in Figure 3.

First, the variables were offset by setting the location of the ankle markers as midpoints as the patient stands on the front part of the level floor.

Next, we eliminated the anthropometric differences between subjects using the distance between shoulder and ankle markers using Equation (1):


(1) $$\begin{array}{r} p_{i}^{{}^{\prime }}=\frac{p_{i}}{l_{1}+l_{2}+l_{3}},i=1,2,...,n, \end{array}$$

where *p*_*i*_ is the *i*-th X/Y/Z-axis position of the kinematic variable; $p_{i}^{{}^{\prime }}$ is the *i*-th X/Y/Z scaled marker position; *l*_1_ is the average distance between the shoulder and hip markers; *l*_2_ is the average distance between the hip and knee markers; and *l*_3_ is the average distance between the knee and ankle markers. *n* is the number of kinematic variables, and the value of *n* was set to 33.

Next, we used a double-pass, second-order Butter-worth low-pass filter with a cutoff frequency of 12 Hz to filter the *p*′ data. Thus, the noise arising from changes in the orientation of the subject's body and other factors during measurement (Abdulhay et al., 2018) were removed.

**Figure 4:**
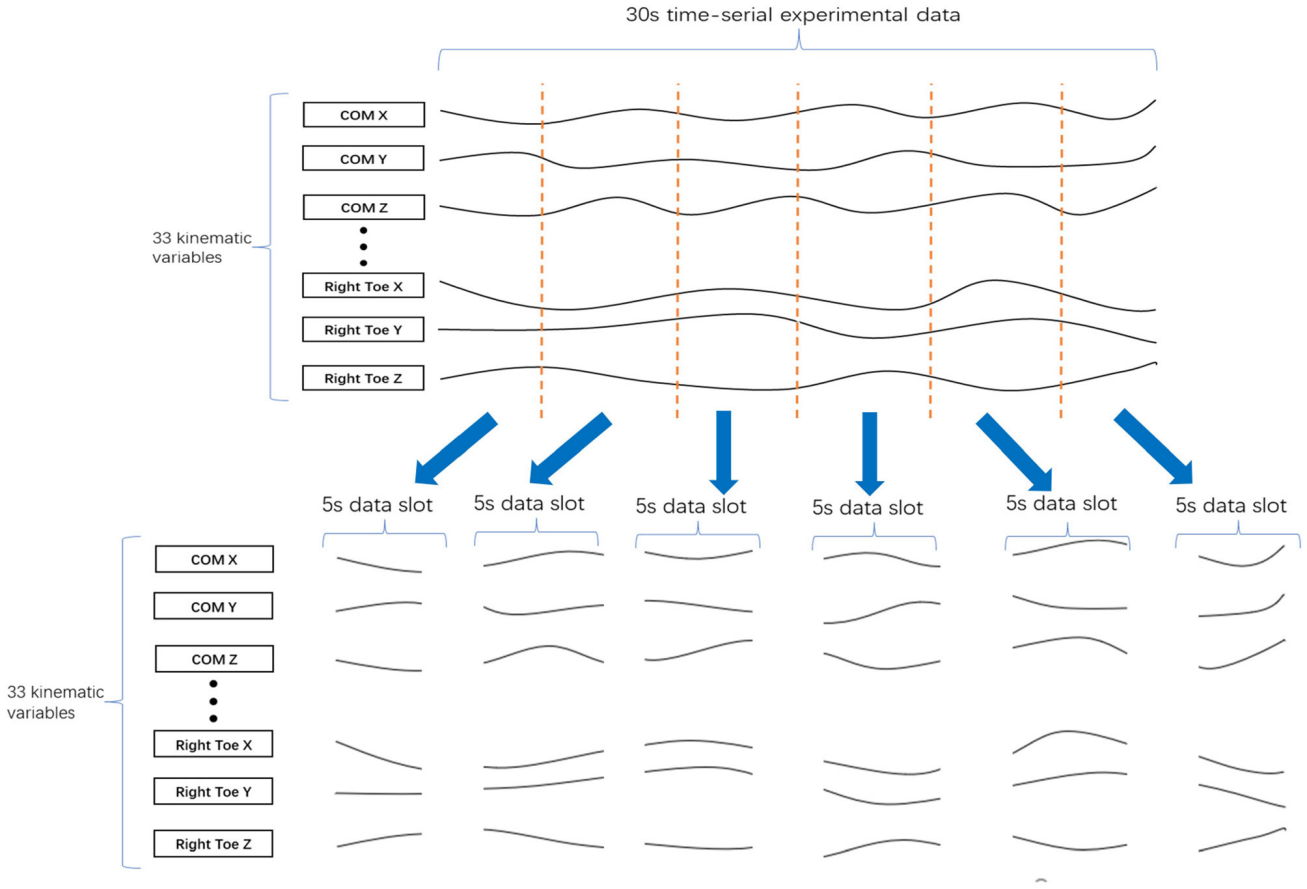
Division of 30 s of data into six 5-s data slots. A 30 s set of experimental data with 33 kinematic variables was divided into six of 5-s data slots, where each data slot has 33 kinematic variables.

Finally, to increase the data set size for each cluster, we divided every 30-s data sample into several data slots *t* seconds in length. For example, the division of a 30-s data sample into six 5-s data slots is shown in Figure 4. To determine the best value of *t*, we tested a range (*t* = 3, 5, 6, and 10) by evaluating the clustering results.

### 2.3. Clustering

To find the postural-sway patterns of the post-stroke patients and to identify the relationships between the patient body displacement and their different characteristics (e.g., age, days after onset, and hemiplegia side), we used the multivariate time-series (MTS) clustering method. In a related field, numerous MTS methods have been explored (Montero and Vilar, 2014; Brandmaier, 2015; Genolini et al., 2015; Sardá-Espinosa, 2019).

**Figure 5:**
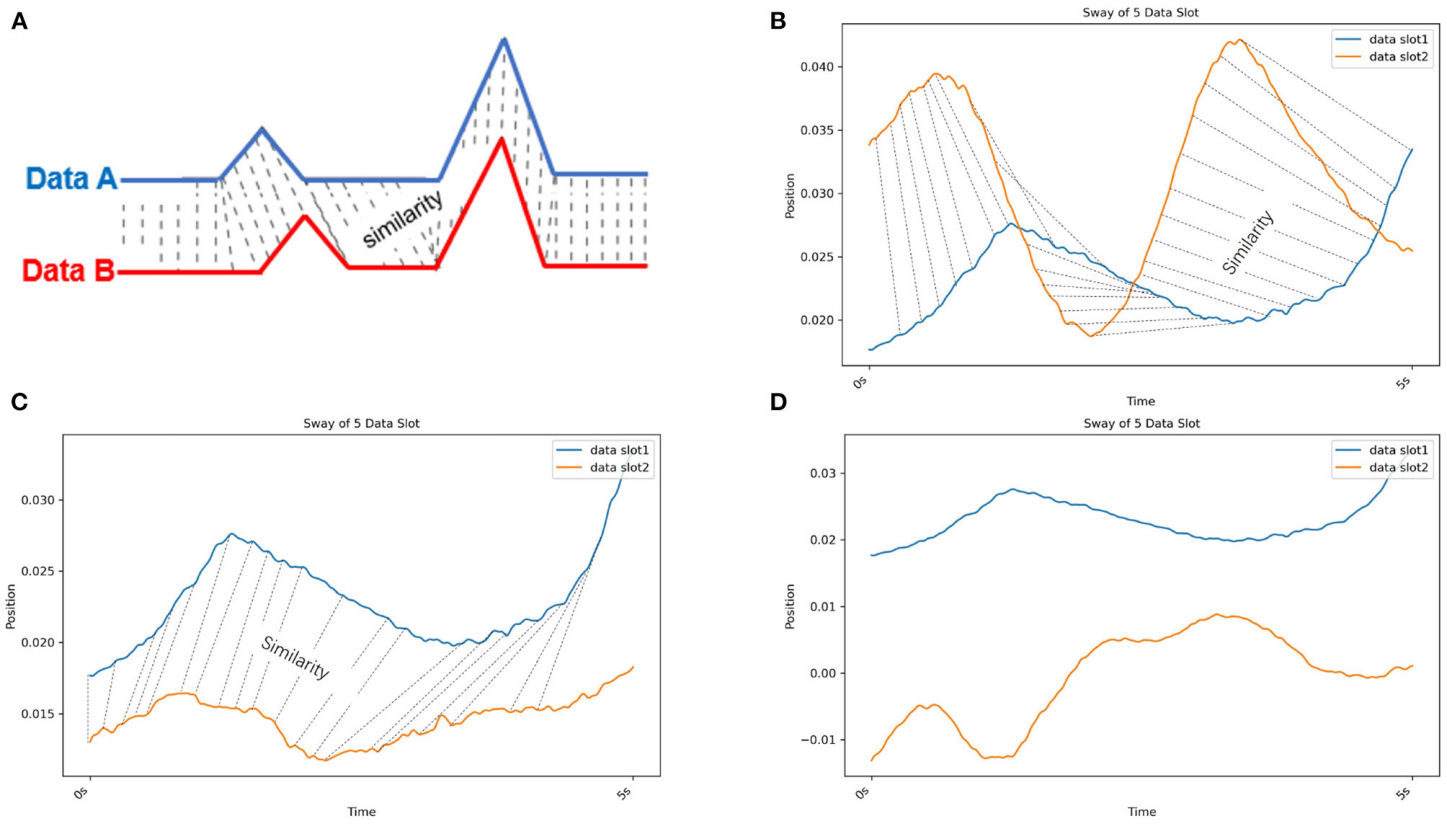
**(A)** Dynamic time warping (DTW). DTW compares the similarity between two temporal sequences (Data A in blue and B in red) that can vary in speed. **(B)** Dynamic time warping (DTW) with real postural sway data from the same individual. The blue and orange lines are the COM X data of different data slots from CH2. **(C)** Dynamic time warping (DTW) with real postural sway data from different patients in the same cluster. The blue line is the COM X data of a data slot from CH2. Orange line is the COM X data of a data slot from CH3. **(D)** Dynamic time warping (DTW) with real postural sway data from different patients in different clusters. The blue line is the COM X data of a data slot from CH2. The orange line is the COM X data of a data slot from CH5. Because the similarity of these two data slots is low, no similarity lines could be drawn.

As one method of MTS clustering, partitioning clustering with dynamic time warping (DTW) clustering is used to evaluate the similarity of different data slots (Malik and Lai, 2017; Rybarczyk et al., 2018). In Figure 5, the DTW compares the similarity between two temporal sequences (Data A and B), which may vary in speed. The DTW is used for temporal sequences, such as video, audio, and graphics data. Moreover, compared with other MTS clustering methods (e.g., a permutation distribution cluster), DTW provides faster calculations (Montero and Vilar, 2014; Sardá-Espinosa, 2019). Therefore, we used the DTW method to evaluate the similarity of data slots from different patients. To illustrate the similarity exhibited by data slots of 5 s in length in the case of postural sway, we present Figures 5B–D which show the similarity in 5-s slot data taken from individuals as well as from different patients. Moreover, we can evaluate the sway amplitude and frequency measured from the data slot as its similarity feature via DTW. Furthermore, this method calculates the distance between all points in the data; hence, the smaller the gap, the closer the match. The DTW could work well on the 33 kinematic variables because the postural sway of human is periodic (Giveans et al., 2011). All periods of sway data are similar. Thus, we assumed they can be detected by DTW. Moreover, to avoid the cut off of such periods, based on the period, we divide the 30 s of data into *t*-s data slots. Another researcher also used DTW to detect hip sway (Cuntoor et al., 2003).

After assessing the similarity between different slots, DTW clustering divides data slots into *K* clusters, and each cluster has a centroid. Here, the cluster implies a group wherein the data slots are more similar than those in other groups. Moreover, the centroid is the cluster center. We attempted two different centroid calculation methods. One is the partition-around-medoid (PAM) method, and another is DTW barycenter averaging (DBA) (Sardá-Espinosa, 2019).

**Figure 6:**
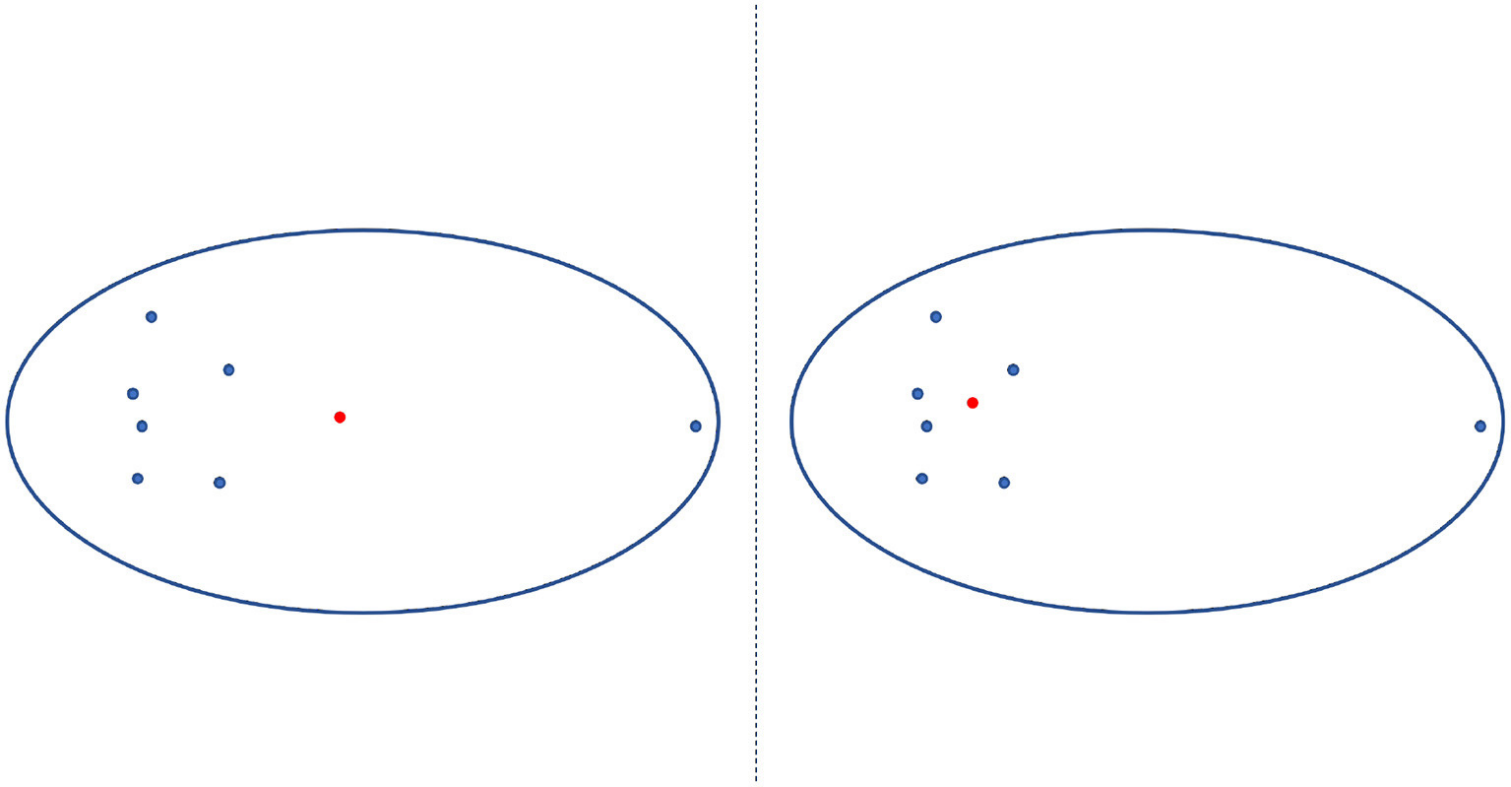
Mean center vs. medoid center. The mean center is indicated by the red dot in the left panel and differs the medoid center (indicated by the red dot in the right panel), which is the most central object in the clusters with the smallest sum of distances from other data.

The PAM is a method to find *K*-medoids point of clustering (Mannor et al., 2011). First, PAM selects *K* representative medoids (the most central clusters) to construct an initial cluster. Then, it continuously changes the medoids to find a better cluster representative with more significant reductions in distortion function. In each iteration, the set of best medoids for each cluster forms a new respective medoid. As shown in a 2-dimensional example of Figure 6, the medoid center in the red dot in the right figure is the most central object in the clusters with the smallest sum of distances from other data, which differs from the mean center in the red dot in the left figure.

**Figure 7:**
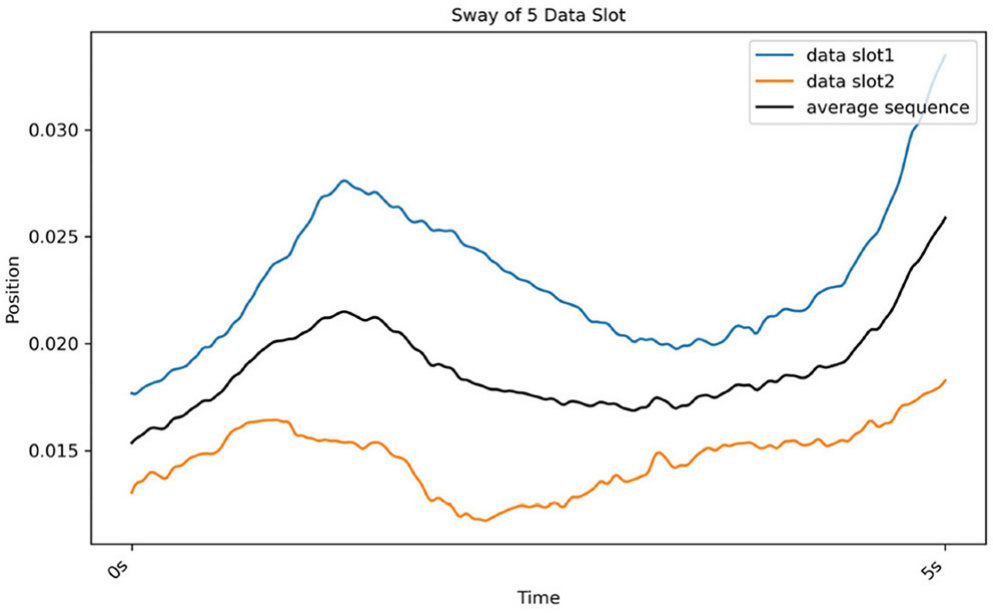
Results of DBA, which gradually determines the average sequence (black line) of two sequences (red line from CH2 and blue line from CH3).

The DBA refines another method of finding the *K*-medoids method. Here, medoids were defined as an average sequence of sets of sequences. The cluster was divided based on the distance between the average sequence and sets of sequences (Petitjean et al., 2011). Real postural sway (marker) data is used to further illustrate this process in Figure 7.

We present the set of 5-s data slots as an example. The input data consisted of 186 sets of 33 × 300-dimensional vector data representing the standardized X/Y/Z-axis positions of 10 markers and a COM from 31 sets of 30 s of experimental data. We had 31 of 30 s experimental data. Every 30 s of data was divided into six slots of 5-s data. Hence, we had a total of 31 × 6 (186) sets of (33 × 300)-dimensional vector data. Because the measurement sampling frequency was 60 Hz for 30 s, for 5 s data, the number of columns is 60 Hz × 5 s (300). The DTW clustering compared each vector of one data slot and its corresponding vector of another. We randomly initialized the centroid of the cluster, and to avoid the effect of random errors in a centroid and to choose the best clustering solution, we repeated the process 10 times for the data set.

Afterwards, the method of determination of the best *K* clusters was introduced. First, to avoid the effect of random centroid behaviors and to choose the best clustering solution, we repeated the process 10 times for each data slot. Next, we evaluated the cluster solution using three cluster indices, including the Davies-Bouldin (DB) index (Davies and Bouldin, 1979), the Calinski-Harabasz (C-H) index (Caliñski and Harabasz, 1974), and the Dunn (D) index (Dunn, 1974), separately. These three indices evaluated the minimum value of the product of mean and the SD of the intra-cluster gap.

The DB is shown in Equation (2), where *K* is the number of clusters. In cluster *i*, δ_*i*_ is the mean gap between data units to their cluster centers, *c*_*i*_. In cluster *j*, δ_*j*_ is the mean gap between all data units to their cluster centers, *c*_*j*_. *d*(*c*_*i*_, *c*_*j*_) is the gap of cluster centers, *c*_*i*_ and *c*_*j*_. The best cluster solution has the minimum DB value.


(2) $$\begin{array}{r} DB=\frac{1}{K}\overset{K}{\underset{i=1,i\neq j}{\sum }}max\left(\frac{\left(\delta_{i}+\delta_{j}\right)}{d\left(c_{i},c_{j}\right)}\right). \end{array}$$

The C-H index is defined Equation (3), where K is the number of clusters and *N* is the volume of the data set. The BGSS indicates the sum of squares of the partition between clusters, and WGSS represents the sum of squares of the partition within a cluster. The best cluster result has the biggest indicator value.


(3) $$\begin{array}{r} C-H=\frac{\left(N-K\right)}{\left(K-1\right)}\times \frac{BGSS}{WGSS}. \end{array}$$

In the cluster, the D index is defined as value calculated by dividing the smallest distance (*d*_*min*_) within the cluster to the biggest distance (*d*_*max*_), as shown in 4:


(4) $$\begin{array}{r} D=\frac{d_{min}}{d_{max}}. \end{array}$$

### 2.4. Parameter Distribution Calculation

After obtaining clustering solutions of the *t*s data slot and the *K* cluster, we analyzed the sway features of each kinematic variable of *t*-s data slots between clusters by applying a *post-hoc* test.

Here, based on previous research (Petri, 2002; Paillex and So, 2005; Abdulhay et al., 2018), we calculated three kinds of sway feature parameters for each kinematic variable of *t*-s data slots: amplitude, standard deviation (SD), and sway frequency. Amplitude is defined as the gap between maximum and minimum values of one kinematic variable in one *t*-s data slot. The SD is defined as the standard deviation of one kinematic variable in one *t*-s data slot. Sway frequency is defined as the frequency value corresponding to the first most prominent peak in frequency domain map by the fast Fourier transform.

Before implementing the *post-hoc* test, we first determined the *post-hoc* test method by observing distribution and the homogeneity level of parameters using Shapiro-Wilk (Mohd Razali and Bee Wah, 2011) and Bartlett's tests (Tobias and Carlson, 1969). If the parameter data follow a normal distribution and display homogeneity of variance, we can use Turkey-Kramer *post-hoc* method. Otherwise, we use a pairwise Wilcoxon test (Pohlert, 2014) with a Benjamini-Hochberg *p*-value adjustment method. Therefore, we performed a *post-hoc* test to find which cluster pairs were significant to each parameter.

We consider the case of the left shoulder marker when the data slot is 5 s and *K* = 4 as an example. First, based on the clustering result, we grouped the 186 data slots into four groups. Then for each group, we extracted the 5-s left-shoulder markers' *x*, *y*, and *z* values from each slot. Then, we calculated amplitude, SD, and sway frequency for each kinematic variable in each data slot. Finally, we implemented the *post-hoc* to find the significant kinematic variables for discussion.

## 3. Results

### 3.1. General Cluster Performance

In this work, we used two models, DTW-PAM and DTW-DBA. Based on the cluster validity evaluation, we compared the cluster results in which the data-slot time, *t*, was in the range of 3, 5, 6, and 10 s; *K* was in the range of 3, 4, and 5; and the method was DTW-PAM or DTW-DBA, as shown in Tables 2, 3. Then, we found that the DTW-PAM model of the *t* = 5-s data slot with *K* = 4 was better than the other results. Hence, we inferred from the clustered index that there was a difference in the standing postures of post-stroke patients. Then, only the detailed solution of DTW-PAM was introduced.

**Table 2:** DTW-PAM result.

**Data slot time t**	**Cluster number K**	**Cluster validity**		
		**C-H index**	**DB index**	**D index**
	3	98.70	1.73	0.03
3 s	4	76.70	1.63	0.02
	5	63.78	1.29	0.03
	3	58.70	1.55	0.06
5 s	4	60.84	1.45	0.01
	5	45.36	1.19	0.06
	3	47.90	1.76	0.09
6 s	4	34.78	2.37	0.02
	5	35.88	1.42	0.03
	3	27.89	1.85	0.22
10 s	10	18.15	1.30	0.05
	5	19.87	2.19	0.03

**Table 3:** DTW-DBA result.

**Data slot time t**	**Cluster number K**	**Cluster validity**		
		**C-H index**	**DB index**	**D index**
	3	103.38	1.60	0.04
3 s	4	83.89	1.69	0.04
	5	83.17	1.53	0.04
	3	63.80	1.57	0.24
5 s	4	53.48	1.49	0.04
	5	40.92	1.10	0.24
	3	52.39	1.98	0.06
6 s	4	43.24	1.45	0.09
	5	37.20	1.24	0.11
	3	28.32	1.56	0.08
10 s	4	24.44	1.72	0.09
	5	23.17	1.35	0.27

Finally, based on the clustering result of the *t* = 5-s data slot with *K* = 4 on the DTW-PAM model, we observed and calculated the median value of days after onset, age, and disease-type percentage from the first to the fourth clusters, as shown in Table 4.

**Table 4:** Characteristics of cluster under data time slot = 5 s, *K* = 4.

**Cluster index**	**Days after onset**	**Age**	**Disease-type percentage**
Cluster 1	1,108	54	CH: 0.6, CI: 0.4
Cluster 2	520	63	CH: 0.75, CI: 0.25
Cluster 3	340	54	CH: 0.95, CI: 0.05
Cluster 4	109	59	CH: 0.43, CI: 0.57

### 3.2. Parameter Distribution Analysis

**Figure 8:**
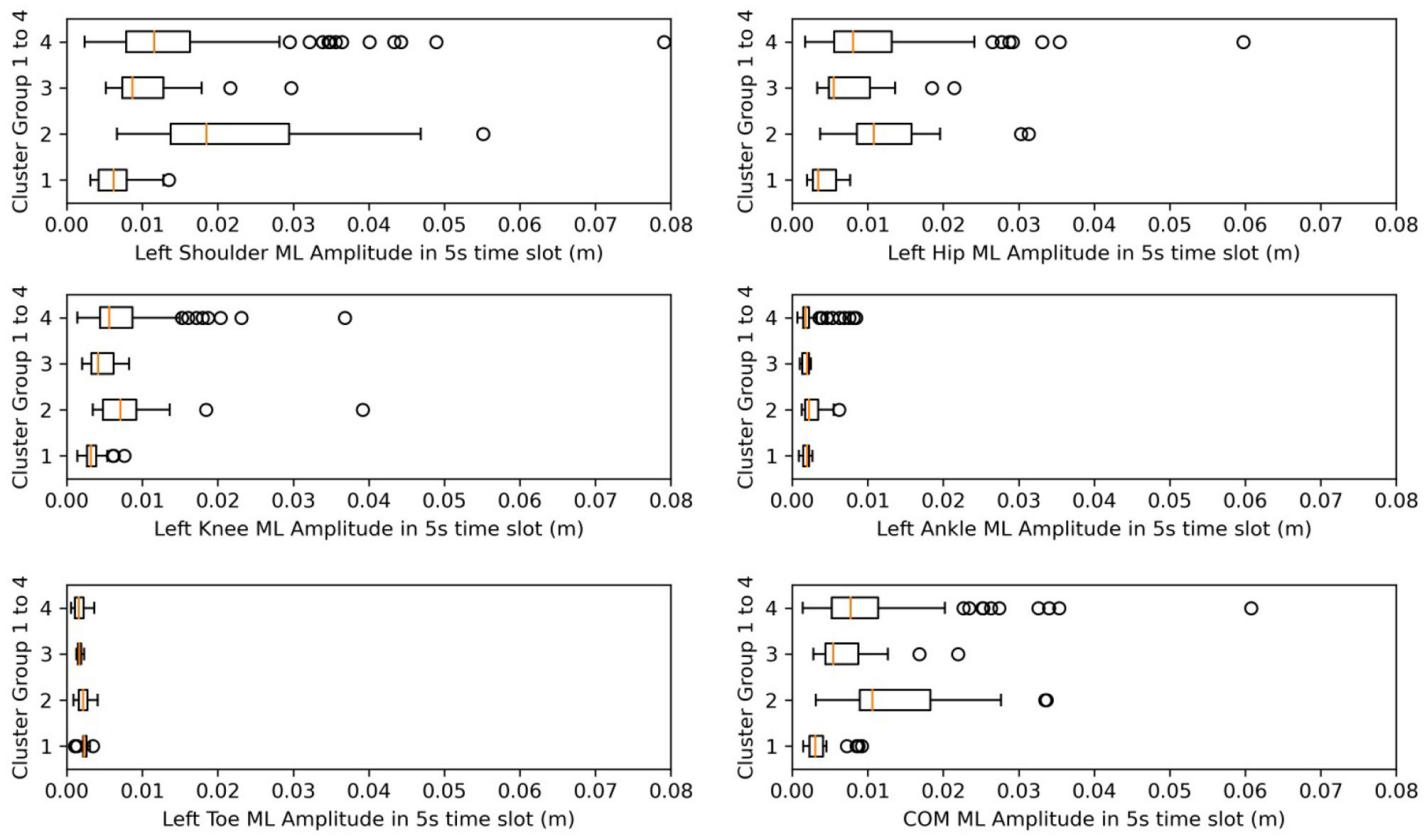
Box plots for amplitude in ML. X-axes represent the amplitude after normalization in the ML direction.

**Figure 9:**
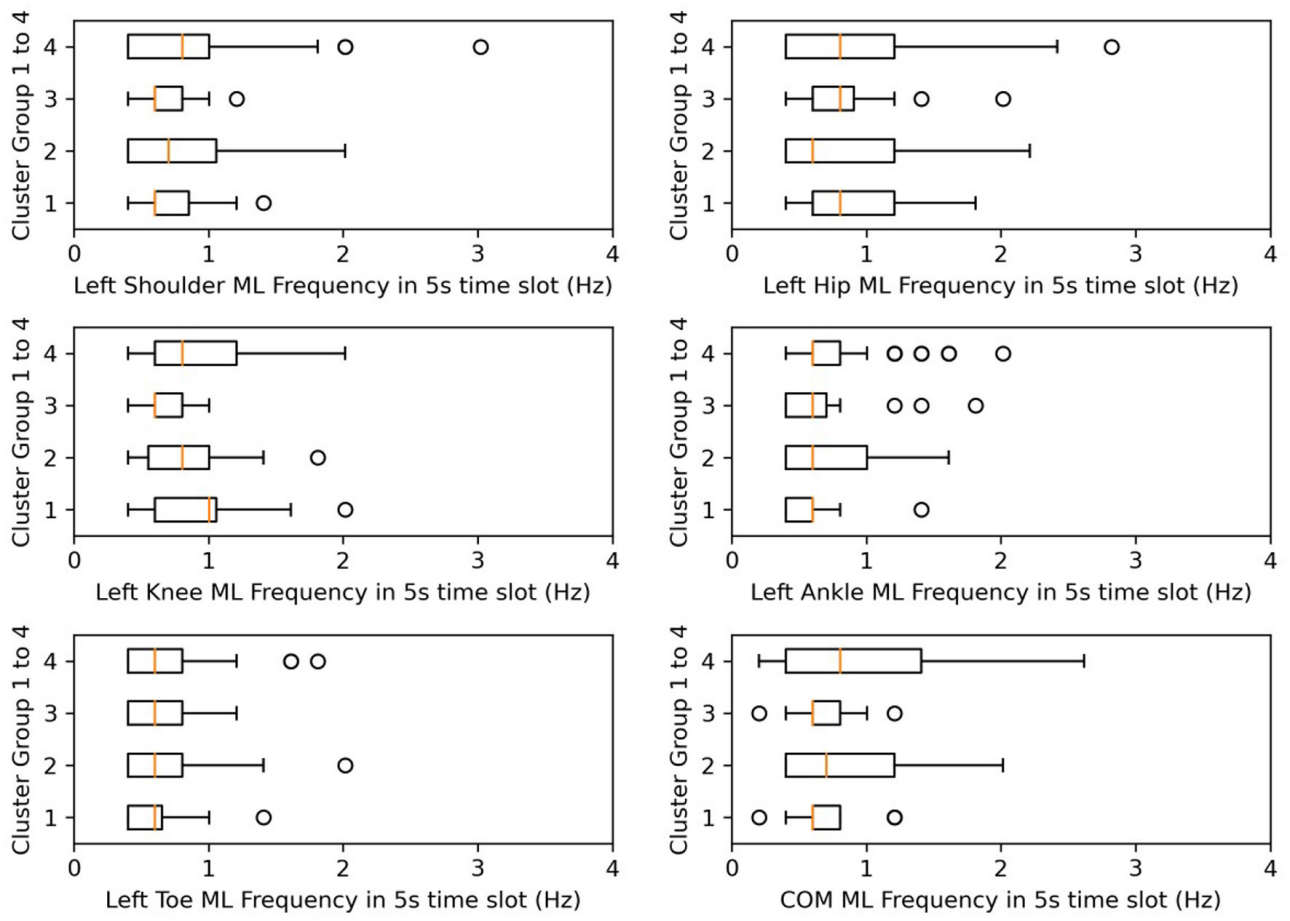
Box plots for frequency in ML. X-axes represent the postural-sway frequency in the ML direction.

To analyze the sway features of each kinematic variable of *t*-s data slots between clusters, first, using the Shapiro-Wilk test, we found that the parameters did not follow a normal distribution and displayed homogeneity of variance. Therefore, we used the pairwise Wilcoxon test (Pohlert, 2014) to perform the *post-hoc* test to find clusters representing significant differences. In Table 5, the *post-hoc* test subjects and results are listed. For each axis of each body on the left or right side (indexed from 1 to 33), we performed a *post-hoc* test to determine which cluster pairs had significant differences. The result shows that the differences between clusters are mainly explained by amplitude and SD. In Table 5, the contribution of the shoulder, hip, knee, and COM variables that are particularly significant are colored blue. As a result, we present Figures 8, 9, whose *x*-axes represent the ML amplitude after normalization and postural-sway frequency, respectively. The kinematic variables of the three axes (*x, y, z*) are likely to be strongly correlated with each other in Table 5. Thus, only the values of the index in the X-axis (ML) are shown. The left and right sides are similar; only the left side is shown.

**Table 5:** *Post-hoc* test results for each axis of each body on the left or right side to determine significant kinematic variables.

**Amplitude**					**SD**					**Frequency**				
**Index**	**Part**	**L/R**	**Axis**	***Post-hoc* test result**	**Index**	**Part**	**L/R**	**Axis**	***Post-hoc* test result**	**Index**	**Part**	**L/R**	**Axis**	***Post-hoc* test result**
1	COM	None	X	1&2, 1&3, 1&4, 2&3, 2&4	1	COM	None	X	1&2, 1&3, 1&4, 2&3, 2&4	1	COM	None	X	None
2	COM	None	Y	1&2, 1&3, 1&4, 2&3, 2&4	2	COM	None	Y	1&2, 1&3, 1&4, 2&3, 2&4	2	COM	None	Y	None
3	COM	None	Z	1&2, 1&3, 1&4, 2&4, 3&4	3	COM	None	Z	1&2, 1&3, 1&4, 2&4, 3&4	3	COM	None	Z	None
4	Shoulder	Left	X	1&2, 1&3, 1&4, 2&3, 2&4	4	Shoulder	Left	X	1&2, 1&3, 1&4, 2&3, 2&4	4	Shoulder	Left	X	None
5	Shoulder	Left	Y	1&2, 1&3, 1&4, 2&3, 2&4	5	Shoulder	Left	Y	1&2, 1&3, 1&4, 2&3, 2&4	5	Shoulder	Left	Y	1&4
6	Shoulder	Left	Z	1&2, 1&3, 1&4, 2&3, 2&4	6	Shoulder	Left	Z	1&2, 1&3, 1&4, 2&3, 2&4	6	Shoulder	Left	Z	None
7	Shoulder	Right	X	1&2, 1&3, 1&4, 2&3, 2&4	7	Shoulder	Right	X	1&2, 1&3, 1&4, 2&3, 2&4	7	Shoulder	Right	X	None
8	Shoulder	Right	Y	1&2, 1&3, 1&4	8	Shoulder	Right	Y	1&2, 1&3, 1&4	8	Shoulder	Right	Y	3&4
9	Shoulder	Right	Z	1&2, 1&3, 1&4, 2&4	9	Shoulder	Right	Z	1&2, 1&3, 1&4, 2&4	9	Shoulder	Right	Z	None
10	Hip	Left	X	1&2, 1&3, 1&4, 2&3, 2&4	10	Hip	Left	X	1&2, 1&3, 1&4, 2&3	10	Hip	Left	X	None
11	Hip	Left	Y	1&2, 1&3, 1&4, 2&3, 2&4	11	Hip	Left	Y	1&2, 1&3, 1&4, 2&3, 2&4	11	Hip	Left	Y	None
12	Hip	Left	Z	1&2, 1&3, 1&4, 2&4	12	Hip	Left	Z	1&2, 1&3, 1&4, 2&4	12	Hip	Left	Z	None
13	Hip	Right	X	1&2, 1&3, 1&4, 2&3	13	Hip	Right	X	1&2, 1&3, 1&4, 2&3	13	Hip	Right	X	None
14	Hip	Right	Y	1&2, 1&3, 1&4, 2&3, 2&4	14	Hip	Right	Y	1&2, 1&3, 1&4	14	Hip	Right	Y	None
15	Hip	Right	Z	1&2, 1&3, 1&4	15	Hip	Right	Z	1&2, 1&3, 1&4	15	Hip	Right	Z	None
16	Knee	Left	X	1&2, 1&3, 1&4, 2&3, 3&4	16	Knee	Left	X	1&2, 1&3, 1&4, 2&3, 3&4	16	Knee	Left	X	None
17	Knee	Left	Y	1&2, 1&3, 1&4	17	Knee	Left	Y	1&2, 1&3, 1&4	17	Knee	Left	Y	None
18	Knee	Left	Z	1&2, 1&3, 1&4, 2&4	18	Knee	Left	Z	1&2, 1&3, 1&4, 2&4	18	Knee	Left	Z	1&2, 2&4
19	Knee	Right	X	1&2, 1&3, 1&4, 2&3	19	Knee	Right	X	1&2, 1&3, 1&4	19	Knee	Right	X	None
20	Knee	Right	Y	1&2, 1&3, 1&4	20	Knee	Right	Y	1&2, 1&3, 1&4	20	Knee	Right	Y	None
21	Knee	Right	Z	None	21	Knee	Right	Z	None	21	Knee	Right	Z	None
22	Ankle	Left	X	3&4	22	Ankle	Left	X	None	22	Ankle	Left	X	1&4
23	Ankle	Left	Y	1&2, 2&3, 2&4	23	Ankle	Left	Y	1&2, 2&3, 2&4	23	Ankle	Left	Y	None
24	Ankle	Left	Z	1&2, 2&3, 2&4	24	Ankle	Left	Z	1&2	24	Ankle	Left	Z	None
25	Ankle	Right	X	1&3, 1&4, 2&3, 2&4	25	Ankle	Right	X	1&3, 2&3	25	Ankle	Right	X	1&2
26	Ankle	Right	Y	1&4, 2&4	26	Ankle	Right	Y	None	26	Ankle	Right	Y	None
27	Ankle	Right	Z	1&2, 1&3, 1&4, 2&4	27	Ankle	Right	Z	1&3, 1&4	27	Ankle	Right	Z	None
28	Toe	Left	X	1&3, 1&4, 2&3, 2&4	28	Toe	Left	X	1&3, 1&4, 2&3	28	Toe	Left	X	None
29	Toe	Left	Y	None	29	Toe	Left	Y	None	29	Toe	Left	Y	None
30	Toe	Left	Z	None	30	Toe	Left	Z	None	30	Toe	Left	Z	None
31	Toe	Right	X	1&2, 1&4, 2&3, 2&4	31	Toe	Right	X	1&2, 2&3, 2&4	31	Toe	Right	X	None
32	Toe	Right	Y	1&2, 1&4, 2&4	32	Toe	Right	Y	1&2, 2&3, 2&4	32	Toe	Right	Y	None
33	Toe	Right	Z	None	33	Toe	Right	Z	None	33	Toe	Right	Z	None

*Taking the first line of Amplitude as an example, “1&2” means the kinematic variable COM X was shown to be significant in clusters 1 and 2 by the post-hoc test. The contribution of the shoulder, hip, knee, and COM variables were particularly significant (indicated by blue)*.

Meanwhile, from Table 4, we find that from clusters 1 to 4, the days after onset value decreased. From Figure 8, we observed a pattern from clusters 1, 3, and 4 in which the amplitude increased, meaning that as days from post-stroke onset decreased, the postural-sway amplitude increased.

Then, we created the days-after-onset cluster table to explore the transition pattern of days after onset in CH and CI separately in Table 6, where we used numbers 1–4 and colors from blue to red to represent the clustered data from days after onset. The numbers from 0 to 1,000 indicate the days after onset for each cluster group. We can see that as the days after onset increased, CH patients transited from clusters 4 to 3, to 2, and to 1, in order, whereas for CI patients, they did not show a pronounced transition over time. Moreover, in Table 6, CH3, CI1, CI2, and CI4 was the cluster which was clustered into different clusters, because it is difficult to cluster them perfectly in machine learning. Clusters in which many data slots are clustered are considered to be the main clusters.

**Table 6:** Clustering by days after onset to explore the transition pattern of days after onset in CH and CI.

**Name index**	**Days after onset**	**0**						**100**						**200**	**300**						**400**	**500**						**600**	**700**	**800**	**900**						**1,000**						**1100**					
CH3	51	4	4	4	4	4	4																																									
CH3	58	4	4	4	4	2	2																																									
CH3	65	4	4	4	4	4	2																																									
CH3	79	4	4	4	4	4	4																																									
CH3	93	4	4	4	4	4	4																																									
CH2	122							4	4	4	4	4	4																																			
CH2	129							4	4	4	4	4	4																																			
CH2	136							4	4	4	4	4	4																																			
CH2	143							4	4	4	4	4	4																																			
CH5	315														3	3	3	3	3	3																
CH5	340														3	3	3	3	3	3																
CH5	358														3	3	3	3	3	3																
CH4	520																					2	2	2	2	2																					
CH4	527																					2	2	2	2	2																					
CH4	534																					2	2	2	2	2																					
CH1	1108																																										1	1	1	1	1	1
CH1	1150																																										1	1	1	1	1	1
CI3	69	4	4	4	4	4	4																																									
CI3	80	4	4	4	4	4	4																																									
CI1	74	4	4	4	4	4	4																																									
CI1	88	4	4	4	4	4	4																																									
CI4	91	2	2	2	2	2	2																																									
CI4	108	4	4	4	4	4	4																																									
CI1	109							4	4	4	4	4	4																																		
CI1	176							4	3	1	1	1	1																																		
CI2	132							4	4	4	4	4	2																																		
CI2	147							4	4	1	1	1	1																																		
CI5	992																														4	4	4	4	4	4												
CI5	1007																																				4	4	4	4	4	4						
CI5	1020																																				4	4 4	4	4	4						
CI5	1051																																				4	4 4	4	4	4						

*Numbers 1–4 and colors from blue to red represent the clustered data from days after onset. The numbers from 0 to 1,000 indicate the days after onset for each cluster*.

## 4. Discussion

This study aimed to determine and understand the postural-sway features of post-stroke patients in quiet standing postures. Using DTW-PAM, differences were observed between patient clusters. The markers' amplitude, SD, and frequency indicated that disease-days after onset and disease subtypes (CH or CI) contributed more to postural-sway features than did other features.

After analyzing Table 5, we determined that amplitude had a similar significance performance as the SD, and it had greater significance than did frequency in the clusters, meaning that amplitude and SD were more valuable than the frequency in the clusters. In particular, the differences were more pronounced in the shoulder, hip, and knee. This finding may provide a focus area for post-stroke patient therapy. From Figure 8, we found that the upper parts of limbs (e.g., shoulder, hip, and COM) had significantly larger amplitude values than did the lower parts of limbs (e.g., knee, ankle, and toes). This finding is similar to a previous study that showed that waist sway was more significant than leg sway (Dickstein and Abulaffio, 2000). From Table 4 and Figure 8, we found that as days from post-stroke onset decreased; the postural-sway amplitude increased in clusters 1, 3, and 4. However, the amplitude of cluster 2 did not follow this pattern, which may be due to age effects that there was a relationship between postural sway changing with age (Kim et al., 2010).

Frequency was not significantly different in the *post-hoc* test, but we found that the frequency of postural sway fell in 0.5–1.5 Hz for COM, and from cluster 3, CH subjects of lower age and lower days after onset kept their sway frequencies within 0.6–0.7 Hz. A previous researcher found that frequency of body sway fell in the range of 0.1–0.2 Hz (Koltermann et al., 2019), and because they also found that post-stroke increases sway frequency (Mizrahi et al., 1989), our finding was found to be reasonable.

Furthermore, in Table 6, we observed that CH-patient body postural sway gradually changed as days after onset increased (clusters 4 to 1). Meanwhile, CI-patient body postural sway did not show the same onset-days correlation. Another researcher found that CH patients made more significant recovery gains, although they had more excellent functional (motor) impairments (e.g., standing and walking) than CI patients. They also found that CH patients having the most severe disability improved more than those with CI of comparable severity (Kelly et al., 2003; Katrak et al., 2009). From this knowledge, we assumed that a CH onset-days correlation might emerge from their better recovery ability. This finding may give researchers and practitioners new ideas about sway-pattern changes during post-stroke patient rehabilitation. In addition, Table 6 shows that the group formed of CH3, CI1, CI2, and CI4 were clustered into different clusters. After observing their data plots and analyzing the patient demographic factors, we found they all had fewer days after onset (less than 180 days, subacute phases). During the subacute phases, the stroke patient recovers more noticably and is more unstable in muscle force than in the chronic phase (Kiran, 2012; Chow and Stokic, 2014). Hence, these patients exhibit different sway patterns, even over the same experiment duration, and data from the subacute phase patients is considered to be difficult to cluster. This hence might be reason why different slots for one person were clustered into different clusters.

This study has certain limitations. The first is that the number of subjects was only 10. With more subjects, more calculations and analyses could be performed. The second is that we only considered male patients. We plan to investigate more subjects, including female patients, and analyze their postural-sway characteristics in future research. The third is that we did not perform clustering for the healthy age-matched and young subjects. If we add such subjects, we could compared the postural sway among healthy human and stroke patients to determine which postural-sway characteristics are important, which will add meaning to the study.

## 5. Conclusion

This study evaluated the postural-sway features of post-stroke patients using a motion-capture system to collect standing posture data. After collecting stroke patients' multi-variable motion-capture standing posture data, we processed them into data slots of *t* seconds long. Subsequently, we determined the optimal length of the data slots and number of clusters, and clustered the *t*-s data slots into *K* cluster groups using the DTW-PAM method. Finally, to find the critical kinematic variables, we performed a *post-hoc* test. We found that the shoulder, hip, knee, and COM played essential roles in clustering, and the amplitude of the marker was more helpful than its frequency. Furthermore, we created a days-from-onset clustering table and a box plot of the shoulder, hip, knee, and COM variable amplitudes and frequency separately in ML direction using 5-s data slots. We found that as the days after onset increased, CH patients transited from cluster four to clusters 3, 2, and 1 of a four-cluster solution, whereas for CI patients, they did not show such pronounced transitions over time. The above finding would provide researchers new ideas about sway-pattern changes for post-stroke patient rehabilitation. In the following research, we plan to increase the number of subjects.

## Data Availability Statement

The original contributions presented in the study are included in the article/Supplementary Material, further inquiries can be directed to the corresponding author/s.

## Ethics Statement

The studies involving human participants were reviewed and approved by Fujita Health University, the University Ethics Committee. The patients/participants provided their written informed consent to participate in this study. Written informed consent was obtained from the individual(s) for the publication of any potentially identifiable images or data included in this article.

## Author Contributions

DL conceived the ideas, designed the methodology, performed the programming, and wrote the paper. JO and KK supervised the paper and provided comments on the research directions. MM preformed the experiments and provided study materials. RC and KT reviewed and edited the paper. All authors have read and approve the final manuscript.

## Funding

This work has been partially supported by JSPS KAKENHI 19H05730 and the Mohammed bin Salman Center for Future Science and Technology for Saudi–Japan Vision 2030 at The University of Tokyo (MbSC2030).

## Conflict of Interest

The authors declare that the research was conducted in the absence of any commercial or financial relationships that could be construed as a potential conflict of interest.

## Publisher's Note

All claims expressed in this article are solely those of the authors and do not necessarily represent those of their affiliated organizations, or those of the publisher, the editors and the reviewers. Any product that may be evaluated in this article, or claim that may be made by its manufacturer, is not guaranteed or endorsed by the publisher.
