# Supplementary material for: Evaluation of Postural Sway in Post-stroke Patients by Dynamic Time Warping Clustering
Source: Front Hum Neurosci. 2021 Dec 3;15:731677. doi: 10.3389/fnhum.2021.731677 (PMC8678529; doi:10.3389/fnhum.2021.731677)
Supplement: Supplementary file 1 [file Data_Sheet_1.PDF]

Supplementary Material

1 SUPPLEMENTARY TABLES AND FIGURES

In this part, we provide the sway waveform as a supporting file for 3-, 5-, 6-, and 10-s slots. Here, we extracted COM x-axis data from CH1 patient. Then, we plotted 10 3-s data slots in Figure S1, six 5-s data slots in Figure S2, five 6-s data slots in Figure S3, three 10-s data slots in Figure S4, and 30-s data slots (raw data) in Figure S5.

1.1 Figures

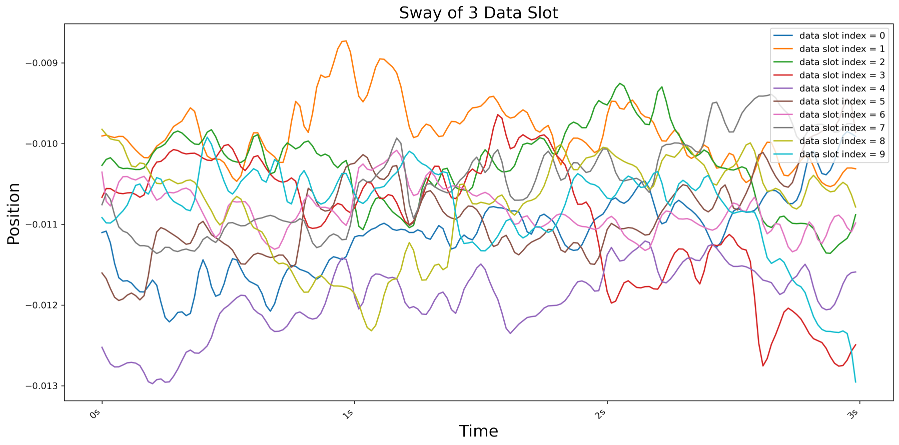

Figure S1. Sway waveform of 3-s data slot

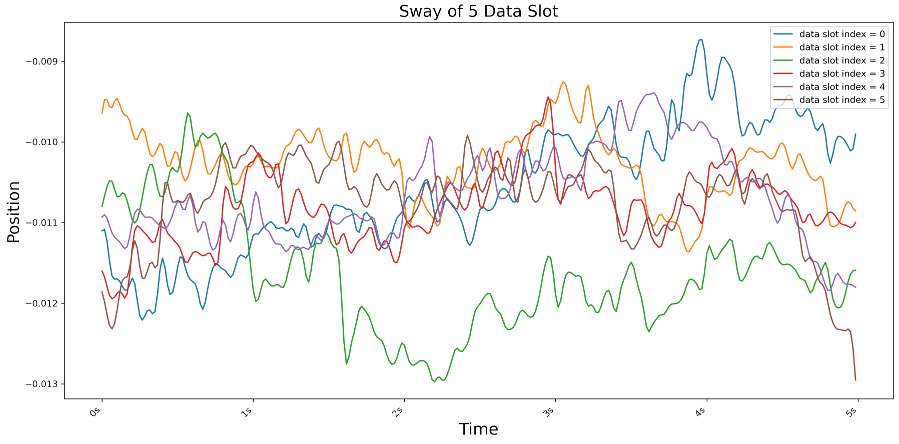

Figure S2. Sway waveform of 5-s data slot

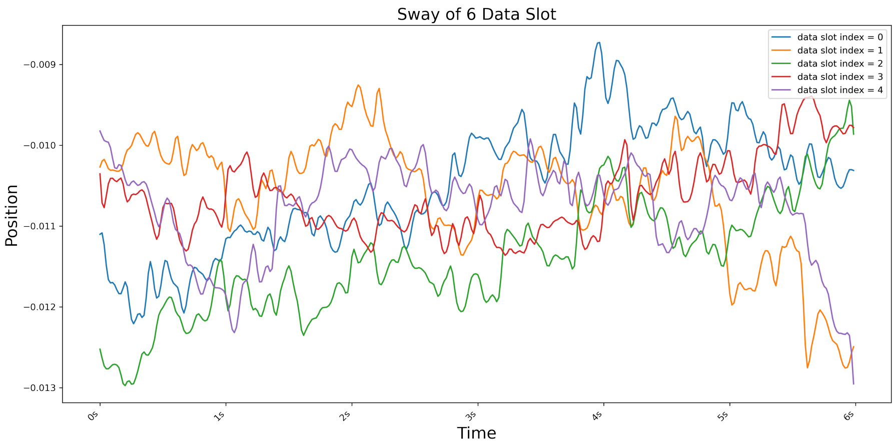

Figure S3. Sway waveform of 6-s data slot

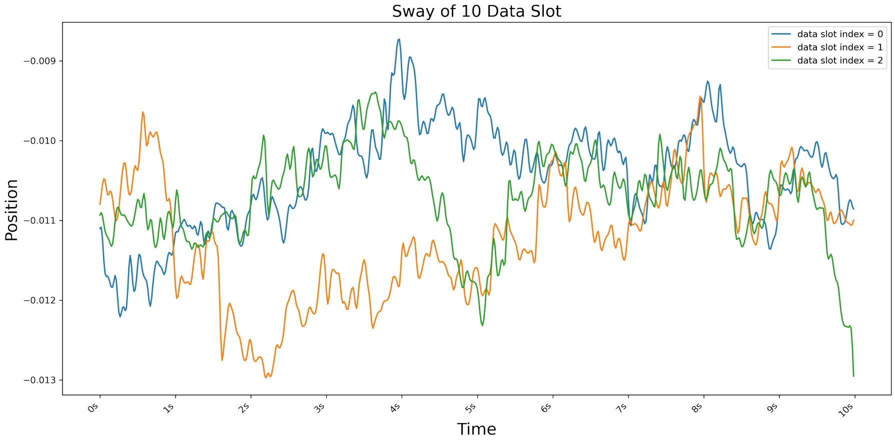

Figure S4. Sway waveform of 10-s data slot

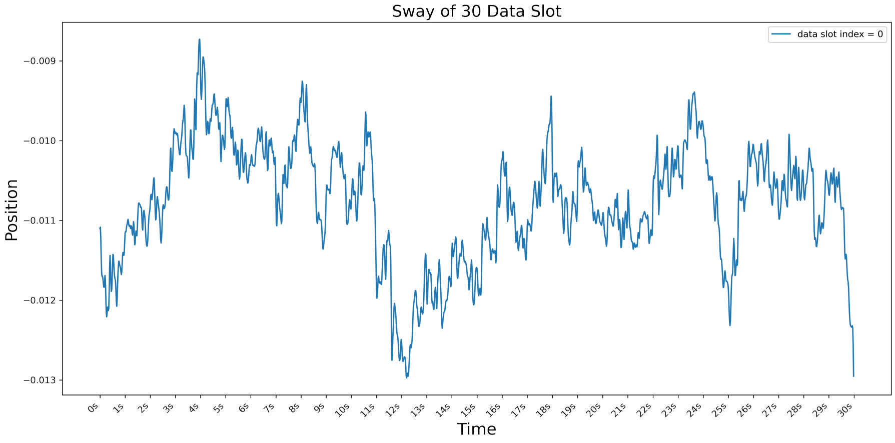

Figure S5. Sway waveform of 30-s data slot
